# Correlation of CpG Island Methylation of the Cytochrome P450 2E1/2D6 Genes with Liver Injury Induced by Anti-Tuberculosis Drugs: A Nested Case-Control Study

**DOI:** 10.3390/ijerph13080776

**Published:** 2016-08-01

**Authors:** Jinling Zhang, Xuebin Zhu, Yuhong Li, Lingyan Zhu, Shiming Li, Guoying Zheng, Qi Ren, Yonghong Xiao, Fumin Feng

**Affiliations:** 1 Hebei Province Key Laboratory of Occupational Health and Safety for Coal Industry, School of Public Health, North China University of Science and Technology, No.57 Jianshe Road, Tangshan 063000, China; xiaoyao_1986@126.com (J.Z.); zxb5060408412@sina.com (X.Z.); 15133912746@163.com (Y.L.); erheyan@163.com (L.Z.); zhengying0918@aliyun.com (G.Z.); qren0819@hotmail.com (Q.R.); kycxyh@tom.com (Y.X.); 2 Department of Clinical Laboratories, Tangshan Tuberculosis Hospital, Tangshan 063000, China; lsm2268909good@163.com

**Keywords:** tuberculosis, anti-TB drug-induced liver injury, methylation, cytochrome P450 2E1, cytochrome P450 2D6, case-control study

## Abstract

This study investigated the role of CpG island methylation of the CYP2E1 and CYP2D6 genes in liver injury induced by anti-TB drugs from an epigenetic perspective in a Chinese cohort. A 1:1 matched nested case-control study design was applied. Pulmonary tuberculosis (TB) patients, who underwent standard anti-TB therapy and developed liver injury were defined as cases, while those who did not develop liver injury were defined as control. The two groups were matched in terms of sex, treatment regimen, and age. In 114 pairs of cases, CpG island methylation levels of the CYP2E1 and CYP2D6 genes in plasma cell-free DNA were found to be significantly correlated with the occurrence of anti-TB drug-induced liver injury (ADLI), with odds ratio (OR) values of 2.429 and 3.500, respectively (*p* < 0.01). Moreover, through multivariate logistic regression analysis, CpG island methylation of the CYP2E1 and CYP2D6 genes in plasma cell-free DNA were found to be significantly correlated with the occurrence of ADLI, with adjusted OR values of 4.390 (95% confidence interval (CI): 1.982–9.724) and 9.193 (95% CI: 3.624–25.888), respectively (*p* < 0.001). These results suggest that aberrantly elevated methylation of CpG islands of the CYP2E1 and CYP2D6 genes in plasma cell-free DNA may increase the risk of ADLI in Chinese TB patients.

## 1. Introduction

With an incidence rate ranging between 5% and 33% [1,2], anti-TB drug-induced liver injury (ADLI) is the main cause of failure or interruption of anti-TB therapy [3]. Consequently, ADLI poses significant challenges to ongoing efforts to control tuberculosis globally.

Anti-TB drugs are metabolized in the liver by phase I and phase II metabolizing enzymes [4,5]. The activities of these enzymes can be influenced by gene polymorphism, which may result in decreased detoxification ability of the liver. Various forms of these enzymes, such as slow acetylation genotype of *N*-acetyltransferase 2, wild genotype of cytochrome P450 (CYP) 2E1, and gene deletion type of glutathione S-transferase M1, have been found to comprise high-risk genotypes for ADLI [6,7,8]. However, the occurrence of ADLI cannot be fully explained by DNA mutation. Many studies have shown that epigenetic factors, especially DNA methylation, may account for individual differences in clinical drug reaction. DNA methylation is the material basis for many phenomena that cannot be explained by DNA variations [9].

Cytochrome P450 is a group of hepatic phase I drug metabolizing enzymes involved in the metabolism and elimination of most drugs in the human body [10]. *CYP2E1* and *CYP2D6* are important members of the *CYP450* family, and polymorphisms of these genes are related to occurrence of ADLI [11,12]. CpG hypermethylation of these genes may also play a key role in the occurrence and progress of ADLI.

CpG island methylation can regulate the efficiency of gene transcription and even lead to its inactivation [13]. If CpG island methylation occurs in genes encoding drug-metabolizing enzymes, it may lead to decline or loss of detoxification activity against toxic metabolites from anti-TB drugs, causing ADLI. A previous study reported that a standard dose of pyrazinamide induced rat liver injury as well as continuous epigenetic change [14], while in another study, 1/3 human dose of isoniazid was found to cause decreased methylation level of whole genomic DNA in rat liver cells and *LINE-1* fragment [15]. Furthermore, low dose of isoniazid has been reported to induce hypermethylation of the *CYP2E1* promoter region in rats [16]. However, the correlation between epigenetic change characterized by methylation and the occurrence of ADLI is not yet supported by a population-level study.

Therefore, this study investigated the relationship between CpG island hypermethylation of *CYP2E1* and *CYP2D6* genes in plasma cell-free DNA of TB patients (under standard anti-TB therapy) and ADLI patients by conducting out a 1:1 matched nested case-control study.

## 2. Materials and Method

### 2.1. Ethics Regulations

The study protocol and informed consent were approved by the Medical Ethics Committee of the Hebei United University (approval no. 10-007). With support from the Tangshan Tuberculosis Hospital, the subjects were informed of the purpose of the study, the routine tests of residual blood samples and the epidemiological investigation. Only those patients who agreed to participate in the study and signed the informed consent form were enrolled.

### 2.2. Sample Size Calculation

The sample size was determined using the Schlesselman formula [17]. With *P*_0_ = 0.25, OR = 2.5, α = 0.05, β = 0.1, the required number of pairs (M) was calculated to be 111 with 81.3% power.

### 2.3. Selection of Experimental Cases and Controls

A total of 114 pairs of TB patients of Han nationality who were hospitalized at Tangshan Tuberculosis Hospital from May 2011 to April 2012 were chosen. Among them, 82 had never been pretreated before and 32 had been pretreated.

*Inclusion criteria:* Cases with normal liver function tests before anti-TB treatment and with liver injury occurring during the follow-up period lasting 6 months. The diagnostic criteria were based on the standard for diagnosis of drug-induced liver injury established by the International Consensus Conference 1990 in Paris [18]. The required alanine aminotransferase (ALT) level was not less than three times the upper limit of the normal value. The basic chemotherapy regimen for new cases was 2S (E) HRZ4HR (S = streptomycin, E = ethambutol, H = isoniazid, R = rifampicin, Z = pyrazinamide; intensive phase for the first 2 months, followed by 4 months of consolidation phase, qd.), the treatment regimen for pretreated cases was 2S (E) HRZ4HR (qod.).

*Exclusion criteria*: 1. Abnormal liver function before anti-TB treatment; 2. Combined with other diseases that may lead to abnormal liver function, such as viral hepatitis, alcoholic liver disease, autoimmune hepatitis, hypoxia, bacteremia, congestive heart failure, and so on; 3. With a history of using other drugs that can cause abnormal liver function, such as chloramphenicol, acetaminophen and chlorpromazine.

*Inclusion of controls*: The controls were TB patients of the same sex with the same place of residence and age difference of less than 5 years, receiving the same regimen of anti-TB chemotherapy and no liver function test abnormalities during 6 months of follow-up.

*Exclusion of controls:* Exclusion criteria for the controls were the same as those for the experimental cases.

### 2.4. Epidemiological Investigation

A unified questionnaire was designed by trained investigators using face-to-face survey. The content of the questionnaire included general information of the subjects, such as sex, age, nationality, occupation, height, weight, educational level and residence, history of tobacco and alcohol use, medical history and medication situation, anti-TB chemotherapy and previous results of liver function tests.

### 2.5. Sample Collection and Extraction of Genomic DNA

A total of 2 mL of EDTA-anticoagulated blood was collected from each subject. The residual blood samples after routine blood test were separated into plasma and blood cells. DNA was extracted using Whole Blood Genomic DNA Purification Mini Kit produced by Beijing Aidlab Biotechnologies Co., Ltd. (Beijing, China). Cell-free plasma DNA was extracted using a cell free DNA extraction kit provided by Beijing Genmagbio Biotechnology Co., Ltd. (Beijing, China). Modification of cell-free plasma DNA was achieved using the EZ DNA Methylation-Gold^TM^ Kit from ZYMO Research Corporation (Irvine, CA, USA). All DNA samples were stored at −80 °C.

### 2.6. Analysis of Gene Polymorphism

PCR primers for the *CYP2E1* and *CYP2D6* genes were selected based on previous studies [19,20,21] and synthesized by Invitrogen Beijing. The universal primers for the *CYP2E1* -1019C/T and -1259G/C polymorphic sites were 5′-CCAGTCGAGTCTACATTGTCA-3′ (forward) and 5′-TTCATTCTGTCTTCTAACTGG-3′ (reverse), respectively. The primers for *CYP2D6* -188C/T and -4268G/C sites were 5′-CCATTTGGTAGTGAGGCAGGTAT-3′/5′-CATGGAGCTCTTCCTCTTCT-3′ (forward) and 5′-CTGTGGTTTCACCCACC-3′/5′-CAAGGGTAACTGACATCTGC-3′ (reverse), respectively. Polymerase chain reaction-restriction fragment length polymorphism (PCR-RFLP) analysis was performed to determine the genotypes of *CYP2E1* -1019C/T, -1259G/C, *CYP2D6* -188C/T, and -4268G/C using RsaI, PstI, HphI and BstEII enzymes, respectively.

### 2.7. Methylation-Specific PCR Amplification

Primers for methylated and unmethylated DNA were designed based on previous studies [16,22] using the Methylation-specific PCR (MSP, a method for analysis of DNA methylation patterns in CpG islands) online primer design software (meth primer). The primers for *CYP2E1*-M and *CYP2E1*-U were 5′-AAATGAATTGATAACGTCGTTAGTAC-3′/5′-TGAATTGATAATGTTGTTAGTATGG-3′ (forward) and 5′-GAAATCAAAATTCTAAACCCGAT-3′/5′-CAAAATCAAAATTCTAAACCCAAT-3′ (reverse), respectively. The primers for *CYP2D6*-M and CYP2D6-U were 5′-TTATTATTTAGATTTTGGGTTTCGG-3′/5′-TTATTATTTAGATTTTGGGTTTTGG-3′ (forward) and 5′-GCTAACCTATTTCATATCCACGAC-3′/5′-CACTCACTAACCTATTTCATATCCACA-3′ (reverse), respectively. 

The PCR amplification system consisted of 10 μL 2× Taq PCR Master Mix, 1 μL each of forward and reverse primers, 2 μL bisulfite-modified DNA template and ultrapure water added to a total volume of 20 μL. The amplification parameters were as follows: Predenaturation at 94 °C for 5 min, denaturation at 94 °C for 30 s, annealing at 60 °C for 30 s, extension at 72 °C for 30 s, 60 cycles, and terminal extension at 72 °C for 3 min. PCR products were analyzed by 2% agarose gel electrophoresis. The samples with bands amplified by the methylation-specific primers was recorded as completely methylated (M); the samples with bands amplified by unmethylation-specific primers was recorded as unmethylated (U); samples with bands amplified by both methylation- and unmethylation-specific primers were recorded as incompletely methylated; both incomplete and complete methylation belonged to the methylation group. 10% DNA samples were randomly selected for repeated tests, and the concordance rate was 100%.

### 2.8. Statistical Analysis

The dataset was established and analyzed using SPSS 13.0 statistical software (SPSS Inc., Chicago, IL, USA). Quantitative data of normal distribution were analyzed by paired *t*-test. Categorical variables were analyzed by paired χ^2^ test or single-factor logistic regression analysis. Then, they were subjected to multi-factor logistic regression analysis, with the occurrence of liver injury after anti-TB chemotherapy as the dependent variable. In two-sided tests, α = 0.05.

## 3. Results

### 3.1. Basic Characteristics of the Cases

During the study, a total of 1312 TB cases were newly diagnosed with sputum smear positive for pulmonary TB and treated in our hospital. A total of 1291 patients finished a six-month follow-up, 21 cases were lost, 164 cases met the diagnostic criteria of ADLI, 114 cases were selected by the treatment time, and 114 cases without liver injury were selected as controls. The general profile of the 114 pairs of cases was as follows: 92 pairs of male cases (80.7%), 22 pairs of female cases (19.3%), 66 pairs of rural cases (57.9%), 48 pairs of urban cases (42.1%). The mean age of the case group was 52.4 ± 17.2 (22–78 years old), whereas the mean age of the control group was 53.1 ± 16.4 (22–79 years old).

Conditional logistic regression showed no statistically significant difference (*p* > 0.05) between the two groups in the distribution of marital status, educational level, occupation, body mass index, drinking and smoking history, and medical history. It was therefore inferred that the two groups were well matched in these aspects.

### 3.2. Correlation with Gene Polymorphism of the Two Drug-Metabolizing Enzymes and ADLI

Table 1 shows that the distribution of genotypes in *CYP2E1* -1019C/T and -1259G/C, *CYP2D6* -188C/T loci was not significantly different between the two groups. The polymorphism of the three sites was not considered to be related to ADLI. The cases with *CYP2D6* -4268G/C mutation had a lower risk of ADLI than those carrying the wild genotype after anti-TB chemotherapy.

### 3.3. Correlation between CpG Island Methylation of Genes of the Two Drug-Metabolizing Enzymes and ADLI

**Figure 1:**
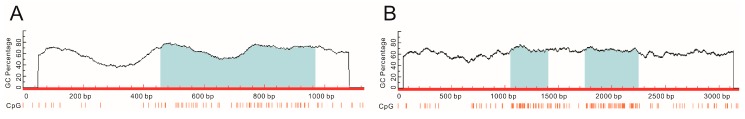
CpG island of *CYP2E1* and *CYP2D6* gene. Note: (**A**) *CYP2E1* gene CpG island; (**B**) *CYP2D6* gene CpG island.

Figure 1 shows that there were CpG islands in the promoter region of *CYP2E1* and *CYP2D6*. It suggests that *CYP2E1* and *CYP2D6* genes may be regulated by DNA methylation.

Table 2 shows that there was a statistically significant difference (*p* < 0.05) in the CpG island methylation level of *CYP2E1* and *CYP2D6* between the case and the control groups. The risk of ADLI was higher in cases with CpG island methylation level of *CYP2E1* and *CYP2D6* by 1.429 and 2.5 times, respectively, compared to those without CpG island methylation after anti-TB chemotherapy. This indicated that CpG island hypermethylation of *CYP2E1* and *CYP2D6* gene is a risk factor for ADLI.

### 3.4. Multivariate Analysis of the Correlation between CpG Island Methylation of Genes of the Two Drug Metabolizing Enzymes and Anti-TB Drug-Induced Liver Injury

In order to control for the confounding effect of polymorphism of the *CYP2E1* and *CYP2D6* genes, the factors with statistical significance in univariate analysis were considered as independent variables, and the occurrence of ADLI was considered the dependent variable in a conditional logistic regression analysis. Table 3 shows that the *CYP2D6* -4268G/C mutation is a genotype protective against ADLI. Thus, CpG island methylation of the *CYP2E1* and *CYP2D6* genes is significantly correlated with the occurrence of ADLI.

## 4. Discussion

This nested case-control study investigated the relationship between CpG island methylation of the genes of hepatic drug-metabolizing enzymes and the occurrence and progress of ADLI from an epigenetic perspective. The results showed that the occurrence of CpG island hypermethylation of the *CYP2E1* and *CYP2D6* genes was significantly different between the case and the control groups. This indicates that TB patients with hypermethylated CpG islands have increased risk of ADLI. CpG island hypermethylation may thus be a new potential risk factor for ADLI besides gene polymorphism. Influence factor analysis also shows that genetic mutation of the drug-metabolizing enzymes is not the only cause of ADLI.

Methylation is recognized as an important mechanism causing tissue and organ inflammation [23]. CpG dinucleotides account for about 10% of the human genome. CpG island is the region with high-density CpG, which is usually hypomethylated. CpG island hypermethylation of gene promoter can affect gene functions and inhibit protein expression by regulating and inactivating gene transcription [24,25]. For example, aberrant methylation of CpG island of the GSTM3 gene promoter is involved in oxidative injury upon liver failure [26]. Furthermore, CpG island methylation of the *BRCA1* gene promoter is associated with breast cancer, and can therefore be used as a biological marker of breast cancer [27]. These studies suggest the importance of CpG island methylation in the regulation of gene transcription.

CpG island hypermethylation of the promoter region of the *CYP2E1* and *CYP2D6* genes leads to ADLI possibly via different pathways involving inhibition of gene transcription. These pathways include direct inhibition of the binding of transcription factors and DNA binding sites, or indirect transcriptional regulation through changes in chromatin conformation, thereby inhibiting the expression of genes of drug-metabolizing enzymes [28,29,30]. By establishing an ADLI model of rat by low-dose isoniazid induction, our previous studies confirmed that methylation level of the whole genome of liver tissue of rats with ADLI was significantly lower than that of the control group [15,16]. Notably, aberrant methylation was also found in the promoter region of the *CYP2E1* gene. However, there has been no population-based study on the correlation between methylation of genomic DNA and ADLI occurrence to date. Therefore, research in this field may provide important clues to early diagnosis of ADLI.

In ADLI, the genes of drug-metabolic enzymes distributed in the liver are released into the blood following the destruction, apoptosis and secretion of liver cells. Then cyclic DNA is either wrapped in nucleosomes or serum proteins [31]. Detection of circulating DNA can be performed for early diagnosis of liver cancer and other malignant tumors [32,33]. Analysis of CpG island methylation of the genes of drug-metabolizing enzymes using circulating DNA in the plasma of TB patients receiving anti-TB chemotherapy can verify its relationship with ADLI.

The present work followed a 1:1 nested matched case-control study design. First-line anti-TB drugs such as isoniazid, rifampicin and pyrazinamide can cause liver toxicity, and may directly or indirectly lead to ADLI. Therefore, it is particularly important that the case group and the control group are matched in relevant aspects. This is also what differentiates the present study from other similar studies and makes the conclusions of the present study more persuasive. Since age and sex are common risk factors of many diseases, these factors were also matched in the present study. The diagnosis of liver injury was based on a globally adopted standard established by International Consensus Conference 1990 in Paris, with the diagnostic level of alanine aminotransferase (ALT) raised to three times the normal limit to facilitate comparison between the case and the control groups.

Drug metabolism in the liver involves a variety of phase I and II drug-metabolizing enzymes. This study was confined to the genes of two drug-metabolizing enzymes belonging to the cytochrome P450 family. There are many other enzymes that were not considered, and further research is needed to determine if they are also related to ADLI. In addition, among the four polymorphic loci considered in this study, polymorphism of only *CYP2D6* -4268 was found to be associated with ADLI. No effects of the three other loci were observed possibly due to the small sample size. Therefore, the sample size needs to be increased in future studies. Furthermore, genetic mutation of hepatic drug-metabolizing enzymes and changes in DNA methylation level may be only some of the factors related to ADLI, and more factors that influence ADLI remain to be identified.

## 5. Conclusions

In conclusion, this study confirmed that CpG island hypermethylation of the *CYP2E1* and *CYP2D6* genes is correlated with the occurrence of ADLI. The adjusted relative risks associated with CpG island hypermethylation of the *CYP2E1* and *CYP2D6* genes based on multivariate analysis were 4.390 and 9.193, respectively. Since both of these values are much greater than 3, it can be inferred that CpG island hypermethylation of the promoter regions of the two genes is strongly associated with ADLI. This discovery not only provides a theoretical basis for in-depth study of the mechanisms of ADLI, but also presents an optimistic prospect for the development of new anti-TB treatment considering the fact that DNA methylation is reversible. For example, expression of the tumor suppressor genes, whose CpG islands are hypermethylated in certain types of cancer, can be restored through demethylation induced by 5-aza-2′-deoxycytidine (a DNA methyltransferase inhibitor) [34,35]. Thus, the mechanism identified in the present study may be exploited for the treatment of ADLI.

## Figures and Tables

**Table 1:** *CYP2E1* and *CYP2D6* genotypes in patients with and without ADLI.

Control	Case		Wald χ^2^	*p* Value	OR (95% CI)
	*CYP2E1* -1019 Mutant Genotype *	*CYP2E1* -1019 Wild Genotype			
*CYP2E1* -1019 Mutant genotype *	10	24	0.667	0.414	0.694 (0.292–1.650)
*CYP2E1* -1019 Wild genotype	30	50			
	*CYP2E1* -1259 Mutant genotype *	*CYP2E1* -1259 Wild genotype			
*CYP2E1* -1259 Mutant genotype *	16	18	2.174	0.140	1.651 (0.731–3.730)
*CYP2E1* -1259 Wild genotype	28	52			
	*CYP2D6* -188 Mutant genotype *	*CYP2D6* -188 Wild genotype			
*CYP2D6* -188 Mutant genotype *	70	18	0.400	0.527	0.707 (0.216–2.312)
*CYP2D6* -188 Wild genotype	22	4			
	*CYP2D6* -4268 Mutant genotype *	*CYP2D6* -4268 Wild genotype			
*CYP2D6* -4268 Mutant genotype *	92	16	6.150	0.013	0.250 (0.084–0.748)
*CYP2D6* -4268 Wild genotype	4	2			

Note: * Mutant genotype consists of both the heterozygous and homozygous mutant genotypes.

**Table 2:** *CYP2E1* and *CYP2D6* gene CpG island methylation in patients with and without ADLI.

Control	Case		Wald χ^2^	*p* Value	OR (95% CI)
	*CYP2E1* CpG Island Methylated *	*CYP2E1* CpG Island Unmethylated			
*CYP2E1* CpG island methylated *	58	14	7.807	0.005	2.429 (1.303–4.525)
*CYP2E1* CpG island unmethylated	34	8			
	*CYP2D6* CpG island methylated *	*CYP2D6* CpG island unmethylated			
*CYP2D6* CpG island methylated *	76	8	9.765	0.002	3.500 (1.595–7.679)
*CYP2D6* CpG island unmethylated	28	2			

Note: * Methylated group consists of both the completely methylated and partially methylated groups.

**Table 3:** Multivariate conditional logistic regression analyses of factors influencing ADLI.

Variables	$\hat{\beta }$	S.E.	Wald χ^2^	*p* Value	OR (95% CI)
*CYP2E1* CpG island methylated	1.479	0.406	13.291	<0.001	4.390 (1.982–9.724)
*CYP2D6* CpG island methylated	2.218	0.528	17.635	<0.001	9.193 (3.264–25.888)
*CYP2D6* -4268G/C mutant genotypes	−2.409	0.779	9.568	0.002	0.090 (0.020–0.414)
